# In Silico Predictions of Ecological Plasticity Mediated by Protein Family Expansions in Early-Diverging Fungi

**DOI:** 10.3390/jof8010067

**Published:** 2022-01-09

**Authors:** Małgorzata Orłowska, Anna Muszewska

**Affiliations:** Institute of Biochemistry and Biophysics, Polish Academy of Sciences, Pawinskiego 5A, 02-106 Warsaw, Poland

**Keywords:** protein family, peptidases, cazymes, earl diverging fungi, cell wall, adaptasome

## Abstract

Early-diverging fungi (EDF) are ubiquitous and versatile. Their diversity is reflected in their genome sizes and complexity. For instance, multiple protein families have been reported to expand or disappear either in particular genomes or even whole lineages. The most commonly mentioned are CAZymes (carbohydrate-active enzymes), peptidases and transporters that serve multiple biological roles connected to, e.g., metabolism and nutrients intake. In order to study the link between ecology and its genomic underpinnings in a more comprehensive manner, we carried out a systematic in silico survey of protein family expansions and losses among EDF with diverse lifestyles. We found that 86 protein families are represented differently according to EDF ecological features (assessed by median count differences). Among these there are 19 families of proteases, 43 CAZymes and 24 transporters. Some of these protein families have been recognized before as serine and metallopeptidases, cellulases and other nutrition-related enzymes. Other clearly pronounced differences refer to cell wall remodelling and glycosylation. We hypothesize that these protein families altogether define the preliminary fungal adaptasome. However, our findings need experimental validation. Many of the protein families have never been characterized in fungi and are discussed in the light of fungal ecology for the first time.

## 1. Introduction

The *Fungi* are a large Kingdom of eukaryotic organisms. They vary in chemical composition, morphological forms, genomic architectures, and ecology. Fungal life strategies range from organic-matter-decomposing saprotrophs to obligate mutualists and harmful pathogens [1]. As fungi are sessile osmotrophic organisms, they owe their lifestyle diversity to the repertoire of secreted proteins (secretome) that play a major role in nutrient degradation and assimilation. The secretome also takes part in protection from the host’s immune system [2] or in moderating relations with mycorrhizal partners [3]. Fungal secretome composition is reflected in their genomes.

Basically, the Kingdom *Fungi* is divided into two main groups. The first group is the subkingdom *Dikarya*, which comprises the evolutionarily young phyla: *Ascomycota*, *Basidiomycota*, and *Entorrhizomycota*. The second group, called early-diverging fungi (EDF) or basal fungi, is composed of the phyla: *Blastocladiomycota*, *Monoblepharidomycota*, *Neocallimastigomycota*, *Chytridiomycota*, *Mucoromycota*, *Zoopagomycota*, and the group *Opisthosporidia* [1]. The majority of mycological studies are focused on *Dikarya*, while research on EDF is rather limited. Due to these trends, the ecological importance and modes of life of basal fungi remain largely unknown [4]. Early-diverging fungi live in a variety of ecological niches. They also occur in the aquatic environment as on land. Terrestrialization brought new challenges to early-diverging lineages and promoted the eventual formation of hyphae. It is a matter of debate regarding which particular group was the first to develop the true hyphae, but it was one of the three labelled as BCZ (*Blastocladiomycota*, *Chytridiomycota*, *Zoopagomycota*) nodes in the seminal work by Kiss and coworkers [5]. EDF adapted to life on land are often preferentially associated with plants and soil (e.g., *Mucoromycota*) or animals (*Zoopagomycota)*, while those living in water or having an intermediate lifestyle are versatile saprotrophs living on pollen, algal and animal parasites (*Blastocladiomycota*, *Chytridiomycota*) [6].

Evolutionary forces shape gene families by means of duplications and losses, leading to complex patterns of gene family distribution on the tree of life [7]. Fungal genomes are no exception here, with a plethora of documented duplications, including whole-genome duplications and massive gene losses [8,9]. The expansion or retrogression of gene families has been described as a general mechanism for adaptation to occupy different niches [10]. We hypothesize that for fungi this statement is especially true when it comes to genes encoding proteins belonging to particular families. Some nodes of the Fungal Tree of Life (FTol) are particularly abundant in losses as an exemplification of selective pressure acting on parasitic (e.g., *Candida*, *Magnaporthe*, *Metarhizium*, *Pneumocystis*), symbiotic (e.g., *Glomus*), or yeast-like organisms (e.g., *Saccharomycotina*, *Malassezia*, *Rhodotorula*) [11]. On the other hand, gene proliferation with gene families related to a particular ecological niche has been documented in plant-degrading saprotrophs and pathogens (white rot and brown rot fungi—laccases, peroxidases, cellulases) [12], entomopathogens (*Metarhizium*, lipases and proteases) [13], and skin-associated fungi (*Malassezia* lipases and proteases) [14]. Moreover, most fungal genome papers describe expanded and contracted protein families with a limited number of protein domains, referred to repeatedly in diverse contexts ranging from pathogenicity (e.g., proteases) to stress tolerance (e.g., SODs—superoxide dismutases). Usually, these protein families (especially proteases and CAZymes) are described in the context of an ecological niche. However, many of these proteins contribute to a range of ecological adaptations often beyond a single primary lifestyle. These dispersed but recurring observations encouraged us to make a systematic study of changeable protein domains in EDF.

In this in silico study, we present 86 protein families putatively associated with the adaptation of early-diverging fungi to their lifestyle. Importantly, these include well known proteins and so far uncharacterized proteins. To achieve this, we mapped 45 EDF and 10 model *Dikarya* proteomes against three widely used protein domain databases: MEROPS [15], CAZy [16], and TCDB [17]. These databases collect information about proteases, carbohydrate-active enzymes (CAZymes), and membrane transport proteins. The main goal of this work is to systematize knowledge in the field of the fungal adaptasome with a special focus on understudied but ecologically vital EDF. Here, we provide a comprehensive list of protein families likely playing a role in fungal adaptation and at the same time a list of gene candidates for phenotyping.

## 2. Materials and Methods

### 2.1. Data Acquisition, Proteome Mapping, and Protein Families Selection

Forty-five predicted EDF and ten Dikarya proteomes were downloaded from NCBI in April 2018. Reference genome sequences are provided in Supplementary Table S4. In order to assess genome completeness, BUSCO with a fungal reference dataset fungi_odb10 was used; Rhizopus oryzae and Aspergillus nidulans were used as Augustus training species for EDF and Dikarya, respectively [18,19]. All proteomes were mapped against CAZY, MEROPS, and TCDB profile libraries. Sets of protein sequences were mapped against MEROPS and TCDB libraries with blastp [20] (e-value threshold of 1 × 10^−10^). For annotation with CAZY profiles stored in dbCAN we used hmmscan [21]. The number of hits to each protein family were used to create tables describing the proteome content for all analysed fungi (Supplementary Table S1). Supplementary Table S2 contains information about the ecology of the analysed fungi derived from the literature (used labels are described below).

Studied organisms were divided into groups depending on the represented lifestyle. We used the following labels to describe these groups: pathogen, an opportunistic pathogen, saprotroph, commensal, mutualist, living in water, living on land, endophyte, associated with plants, and associated with animals. Each organism was assigned to all matching labels. Combining the Supplementary Tables S1 and S2, we obtained datasets containing labelled organisms, and counts of proteins belonging to CAZY, MEROPS, and TCDB protein families. In all of the ecological groups, a median of occurrences for each protein family was calculated. In the next step, groups were compared in pairs to check for median differences between the same protein families in fungi with divergent ecology. To verify that the difference in medians is statistically significant, the Mann–Whitney–Wilcoxon test was used. The test was selected due to the non-normal pattern of distribution (examined using the Shapiro–Wilk test). From every comparison, ten protein families with the greatest difference in median were listed. Protein families that occurred in three or more compared pairs were selected for further analysis.

### 2.2. Sequence Alignment and Phylogenetic Analysis

Sequences belonging to each picked protein family were aligned using the local iterative alignment method in Mafft v. 3.7 (localpair, maxiterate = 100) [22]. All alignments with homologs of MEROPS and CAZY proteins were curated manually. All proteins with deletions in the conserved regions of the enzymatic domain and/or with deletion or invalid substitution in any of the catalytic residues were excluded. As an invalid substitution, we recognized cases in which catalytic residue was replaced with an amino acid whose physical or chemical properties prevented it from performing the function expected for catalytic residue. The curation process was based on published experimental mutations in catalytic residues and/or the properties of given amino acids, and PDB references.

Due to the low specificity of TCDB profiles, alignments related to this database were not curated as described above. Because some proteins were assigned to more than one TCDB profile during mapping, we additionally performed clustering in CLANS [23] for combined sets of families that contained ambiguous homologs.

All alignments were trimmed with TrimAl (model = gappyout) to remove poorly conserved regions [24]. Next, the IQ-TREE 2 [25] was used to predict the best model for phylogenetic analysis (m = MFP), and to build phylogenetic trees based on the Maximum Likelihood (ML) method. ML trees were estimated with aLRT Chi2-based parametric branch supports (alrt = 0) and starting tree search from a random tree (t = RANDOM).

For all analysed protein families, we assessed domain architecture (pfam_scan.pl [26]), protein localization and the presence of signal peptides (WoLF PSORT [27], SignalP v 5.0 [28].

Phylogenetic trees were visualized and annotated with iToL [29].

## 3. Results and Discussion

We identified 86 protein families which are over and underrepresented in EDF depending on ecological features (referred hereafter as the adaptasome). The adaptasome encompasses 19 MEROPS families of peptidases and inhibitors, 43 CAZymes and 24 transporters (Table 1). Several of the selected protein families have well documented roles in fungal biology, for instance, as cell-wall-related enzymes, while others have never been studied in EDF fungi (e.g., endo-1,4-beta-mannosidase GH26), in fungi in general (e.g., LARGE homologs from GT49) or even in Eukaryotes (e.g., CE12 rhamnogalacturonan acetylesterase or unknown peptidase U69 [30]). Depending on the available evidence, we aimed to provide a comprehensive interpretation of the possible contribution of particular protein families to EDF ecological adaptability.

**Figure 1:**
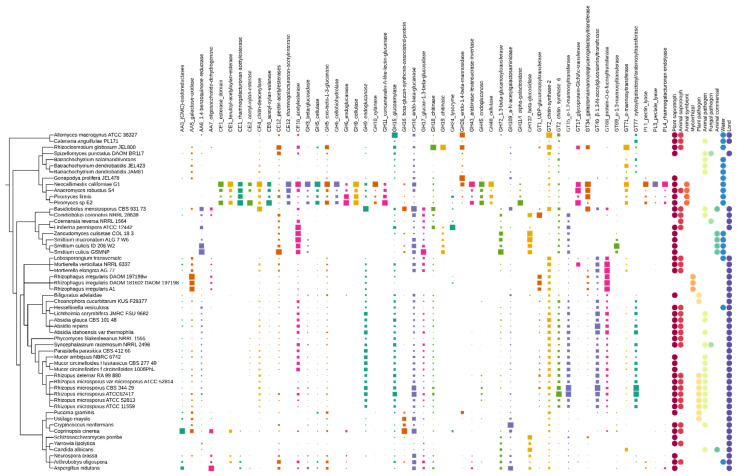
Distribution of adaptasome CaZymes within early-diverging fungi. The presence of proteins belonging to a particular family is marked with squares of size corresponding to their copy number. Fungal ecology features are shown as circles.

In our results (Figure 1) one of the *Rhizopus* representants may draw attention following its outstanding expansion of many families. This is due to an additional round of whole-genome duplication in this strain [31].

### 3.1. Carbohydrate-Active Enzymes Families (CAZymes)

The selected 43 CAZy families have at least 58 biological functions in fungi (Table 1, Figure 1). Most of the CAZymes play a role in either the decomposition of plant origin organic matter or in the remodelling of the fungal cell wall. In addition, we can also distinguish proteins associated with post-translational protein modification, acting as a virulence factor during the infection of animals, and involved in the neutralization of the host’s defence. One of the adaptasome CAZymes putatively acts on lipids. On one hand it is known that glycolipids play a role in diverse interactions; on the other, (glyco)lipid composition varies between fungi, impeding exact function predictions.

The CAZy database is organized according to enzymatic classes into auxiliary activity (AA), carbohydrate esterases (CE), glycoside hydrolases (GH) and glycosyl transferases (GT) and we adhere to this classification.

#### 3.1.1. CAZymes with Auxiliary Activity (AA)

The adaptasome harbours four families of redox enzymes that act in conjunction with other CAZymes (auxiliary activity (AA)). Three out of four AAs are ligninolytic enzymes (AA3, AA5 and AA6). AA3 glucose–methanol–choline (GMC) oxidoreductases are widespread in fungi and catalyse the oxidation of either alcohols or carbohydrates with the concomitant formation of hydrogen peroxide or hydroquinones. Hydrogen peroxide is required for the proper function of lignin degradation peroxidases. This compound may also be provided by copper radical oxidases from the second family AA5 [32]. The third group of AA enzymes associated with lignin utilization is AA6 (1,4-benzoquinone reductases). The activity of this protein was described in the brown rot fungus *Gloeophyllum trabeum*. This fungus uses a quinone redox cycle to generate extracellular Fenton reagent, a key component of its biodegradative system [33]. 1,4-benzoquinone reductases are not only involved in the degradation of aromatic compounds, but also in the protection of fungal cells from reactive quinone compounds. AA6 enzymes are used by entomopathogenic fungi to detoxify quinone secreted by the host. This kind of arms race was observed between the beetle *Tribolium castaneum* and fungus *Beauveria bassiana* [34]. The biggest expansion of AA3 was observed in saprotrophic Basidiomycota—*Coprinus cinerea*. The laccase activity was experimentally demonstrated for this species [35]. All above-mentioned AAs (AA3, AA5 and AA6) were also present in Mucoromycota, as previously shown by Kameshwar and Qin [36], and the expansion of AA5 was observed in arbuscular mycorrhizal fungi Glomeromycotina. However, Glomeromycotina are considered as weak degraders [37].

The AA7 family harbours oligosaccharide flavo-oxidases which can transfer electrons to lytic polysaccharide monooxygenase and, consequently, fuel cellulose degradation [38]. Homologs of AA7 were found in plants, fungi and fungi-like microorganisms. A huge abundance of AA7 genes was revealed in phytopathogenic fungi and *Oomycota* [38]. We also observed an increased occurrence of AA7 homologs in saprotrophic and entomopathogenic fungi.

#### 3.1.2. Carbohydrate Esterases (CE)

Enzymes from the carbohydrate esterases (CE) class occur especially frequently in anaerobic chytrids. Eight out of nine adaptasome CE families are responsible for plant biomass degradation [39,40]. The abundance of carbohydrate esterase enzymes in *Neocallimastigomycota* correlates with their lifestyle as digestive tract symbionts of large herbivores [41]. The families CE2, feruloyl acetylxylan esterase (CE1), and uncharacterized esterases (CE1) show high sequence similarity to bacterial sequences (CE1 feruloyl acetylxylan esterase—ORY83980.1 to MBQ4034677.1 with similarity 57.71%; CE1 uncharacterized esterases—ORY24510.1 to WP_092476704.1 61.76%; CE2—OUM64776.1 to MBQ1538045.1 48.01%) which is consistent with previously documented HGT in this lineage [42]. These transferred genes were duplicated several times and paralogs were retained likely because of environmental requirements.

CE4 chitin de-*N*-acetylases (CDAs) hydrolyse chitin, chitosan, and chitooligosaccharides and are responsible for fungal cell wall remodelling, which is especially important for pathogens during interactions with the host [43,44,45]. Interestingly, CE4 enzymes also play a role in symbiotic interaction between terrestrial fungi and their bacterial symbionts [46]. One might speculate that the abundant fungus–bacteria interactions in the aquatic environment of the gut may also be facilitated by the activity of CE4 proteins.

#### 3.1.3. Glycoside Hydrolases (GH)

Glycoside hydrolases (GH) have an established role in fungal nutrition, particularly in plant biomass degradation and cell wall remodelling [40,47]. The adaptasome encompasses 20 GH families holding 25 different biological functions, inferred from protein databases and the literature. *Neocallimastigomycota*, *Zoopagomycota*, and *Mucoromycota* have lineage characteristic patterns of the expansion and contraction of these 20 GH families. More than half (13) of the identified enzymes are associated with the decomposition of plant biomass. These are xylanase (GH10, GH11), galactosidase (GH114), glucosidase (GH132, GH3), glucoamylase (GH15), glucanase (GH16, GH45, GH6), lysozyme (GH24), mannosidase (GH26), cellulase (GH48, GH5, GH9), and cellobiohydrolase (GH6). Almost all families were previously described in fungi and found to be associated with a saprotrophic lifestyle [48,49]. Mucoromycota are often considered unable to degrade cellulose, but more recent experiments have shown cellulase activity in this lineage [50]. The identification of cellulase expansions in GH48, GH5 and GH9 suggests that the degrading capabilities of Mucoromycota could be broader than expected. Two of the identified CAZyme families have no obvious function—arabinase/levansucrase/invertase (GH43) and lysozyme (GH24) (UniProt-based annotation, prediction was made based on non-fungal organisms). The first enzyme is especially common in anaerobic chytrids and was likely acquired via HGT from bacterial donors (OUM61724.1 to SER94208.1 57.27%). A study on the GH43 family showed the existence of 37 subfamilies, including *Neocallimastigomycota*-specific clades [51], all of them involved in the degradation of plant biomass. Proteins assigned to family GH24 have a Phage_lysozyme (PF00959) domain, which in phage T4 endolysin has lysozyme activity—it degrades host peptidoglycans [52]. Our results show the highest abundance of lysozymes GH24 in *Linderina pennispora*, *Piromyces* sp., and *Coprinopsis cinerea*.

Eight glycoside hydrolase families are involved in cell wall remodelling: galactosidase (GH114), glucosidases (GH17, GH132), chitinases (GH18, GH19), lysozyme (GH24), glucanase (GH5), and glucanosyltransferase (GH72) [47,53]. The cell wall and its remodelling is crucial for survival and strictly associated with interaction with the environment. Remodelling events are important during interaction with a variety of partners. They enable pathogens to interact with plant host tissues [44], the interaction between mycorrhizal partners [54], and immune evasion during infecting animals [55]. Some GH114 members have an additional function: they can disrupt microbial biofilms (e.g., Ega3 from *Aspergillus fumigatus* is an endo-α-1,4-galactosaminidase) [56].

Two changing glycoside hydrolases are probably used by fungi against animal organisms. Chitinase from family GH19 seems to occur only in early fungal phyla such as *Microsporidia*, *Chytridiomycota*, and *Zoopagomycota* (domain distribution in Pfam database). All these fungi are well known from their development on chitin-rich substrates [57]. A described homolog of GH19 in fungi was observed in *Nosema bombycis* and classified as type IV chitinases [58]. Such a distribution may point to early branches of FToL, having their own characteristic workshop for cell wall modification, but not only this. Our results show the expansion of these enzymes in *Zoopagomacota* greater than in other taxa. We hypothesize that GH19 chitinases are used to fight either other fungi in occupied niches (they are known to have antifungal activity [59]) or the chitin armour of the animal host/prey (e.g., insects). This second assumption may also be true for the chytrid *Rhizoclosmatium globosum*—the JEL800 strain was isolated from shrimp chitin bait (JGI website info).

GH132 β-glucosidase, which is also involved in cell wall biogenesis, plays a role as a virulence factor. This enzyme was described as protein Sun41 in *Candida albicans*. Sun41 was proven to promote proper cell wall structure (by having a catalytic role in cell wall modification) and enables biofilm formation. Therefore, Sun41 is required for disseminated infection [60]. The GH132 family has the greatest expansion in *Zoopagomycota* and yeasts.

#### 3.1.4. Glycosyl Transferases (GT)

Most fungal genomic studies refer to the abundance of selected glycosyl transferases (GT) and the adaptasome consistently contains ten GT famlies. Most of these protein families have the greatest expansions in *Mucoromycota* (GT2, GT15, GT49, GT68, GT77).

UDP-glucoronosyltransferase GT1 occurs frequently in *Mucoromycota* and *Entomophtoromycota*. This enzyme was widely described in humans and other mammals, but its function in fungi remains unclear. Due to the composition of the *Mucoromycota* cell wall (high contents of galacturonic acid) we hypothesize that it is associated with the remodelling of this structure [61]. Family GT49 also can be related to the modification of uronic acids. Proteins from this family can be found as β-1,4-glucuronyltransferase in animals, *Capsaspora*, and *Choanozoa*, which indicates their ancestral character. We found representatives of this family predominantly in *Mucoromycota*. It is worth noting that enzymes from GT49 are involved in the pathway of glycosphingolipids biosynthesis. We hypothesize that GT49 takes part in glycocalyx formation [62]. GT49 is known in humans as LARGE protein [63].

The GT2 family contains diverse transferases; among others, the fungal chitin synthases seem to occur in variable copy numbers across taxa. Chitin synthase II is widespread among FToL, and is considered essential for most fungi [64]. All analysed isolates from diverse lineages, except *Kickxellomycotina*, had several copies of this synthase. *Kickxellomycotina* representatives have just a single chitin synthase II and live with arthropods, which happen to have a chitin cuticle. This raises questions regarding whether these two facts are related.

Chitin synthase VI displays a complex domain architecture consisting of Chitin_synth_2 (PF03142), Myosin_head (PF00063), Cyt_b5 (PF00173), and DEK_C (PF08766) domains in various combinations. Three clades of terrestrial fungi show lower copy numbers of chitin synthase VI than the rest: *Mortierellomycota*, *Glomeromycota*, and *Dikarya*.

GT15 α-1,2-mannosyltransferase is involved in the O-glycosylation of the cell wall and secreted proteins in Schizosaccharomyces pombe (encoded by omh1 gene) [65]. In our study, the GT15 family shows expansion in *Zoopagomycota* and *Mucoromycota*, which have a different cell wall glycan ornamentation than *S. pombe*; consequently, the *question* of the mannosylated substrate remains open. GT69 α-1-,3-mannosyltransferase homolog from *Cryptococcus neoformans* was described as part of the polysaccharide capsule’s biosynthesis pathway [66]. In our results, the highest occurrence of GT69 proteins was observed in *Smittium* spp.

Other proteins responsible for the modification of proteins are α-1,3-mannosyltransferase (GT71) and protein-*O*-α-fucosyltransferase (GT68). GT71 has known homologs in yeast, where they are encoded by *mnn* genes [67]. Protein-*O*-α-fucosyltransferase is responsible for protein modification by O-fucosylation. This kind of modification is known from mammals, but to this day there is no information about this process in fungi [68]. GT68 enzymes show outstanding expansion in *Mucoromycota*, which are characterized by high fucose content in their cell wall [61,69]. GT77 also contains proteins probably associated with protein modifications. Among this family, we observed three domain architectures: composed only by Nucleotid_trans (PF03407) domain, or by Nucleotid_trans domain accompanied by GDP_Man_Dehyd (PF16363) or Branch (PF02485) domain.

Two glycosyltransferase families with similar distribution—GT17 (β-1,4-mannosyl-glycoprotein-β-1,4-*N*-acetylglucosaminyltransferase) and GT34 (α-1,2-galactosyltransferase)—are related to the fungal cell wall [47]. These proteins are abundant in *Neocallimastigomycota*, and GT34 also in *Basidiobolus meristosporus*.

Three classes of polysaccharide lyases, pectin lyase (PL1), pectate lyase (PL3), and rhamnogalacturonan-endolyase (PL4), are included in the adaptasome. PL proteins are highly abundant in *Neocallimastigomycota*, but also present in other taxa with plant biomass degradation ability. PL are enzymes typical for fungi, with potential for plant polysaccharide utilization [70].

#### 3.1.5. CAZyme Distribution and Abundance Can Be Linked to the Ecology of Particular EDF Lineages

The anaerobic fungi grouped in *Neocallimastigomycota* show expansions of many protein families in comparison with other fungal taxa. The majority of these duplicated gene families encode enzymes dedicated to the decomposition of plant tissues including AA3, AA5, AA6, CE1, CE12, CE16, CE2, CE6, GH10, GH11, GH114, GH15, GH16, GH26, GH3, GH43, GH45, GH48, GH5, GH6, GH8, PL1, PL3, and PL4. One of the reasons behind the peculiar pattern of CAZymes in *Neocallimastigomycota* is the documented massive horizontal gene transfer from bacteria to these fungi. They thrive with an uncommon life for a fungus, inhabiting an anaerobic environment of the herbivore digestive tract. This particular niche imposes a strong selective pressure on its dwellers. The HGT events allowed anaerobic chytrids to successfully adapt to this ecological challenge [42]. The opposite pattern is present in the case of other chytrids, which presumably lost several families of CAZymes responsible for β-glucan and mannose modification. A similar situation with contracted CAZyme families may be observed in *Saccharomycotina* yeast, and it is probably related to a general tendency towards genome reduction in this lineage. Despite this, yeasts show distinctive expansions of families GH132, GH6, and GH72, which are involved in cell wall modification and biofilm formation. Other fungi with specific expansions of CAZymes are *Bifiguratus adelaidae* and *Rhizoclosmatium globosum*. Losses of CAZymes may also be observed in *Glomeromycotina*, which lack some of the families of plant biomass-degrading enzymes (acetyl-xylan-esterase CE2 and CE6). Such losses are characteristic for plant mycorrhizal symbionts [71]. The phylum *Zoopagomycota* seems to have lost several families of CAZymes which can be linked to the reduced ability to degrade plant biomass. On the other hand, these animal-related fungi have their unique expansions of GH19-related chitinases and AA6 benzoquinone reductase. Discrepancies in CAZyme abundance between *Basidiobolus meristosporus* and *Conidiobolus coronatus* are in line with documented ecological and genomic differences between *Basidiobolales* and *Entomophtorales* (see Figure 1).

### 3.2. Peptidases

**Figure 2:**
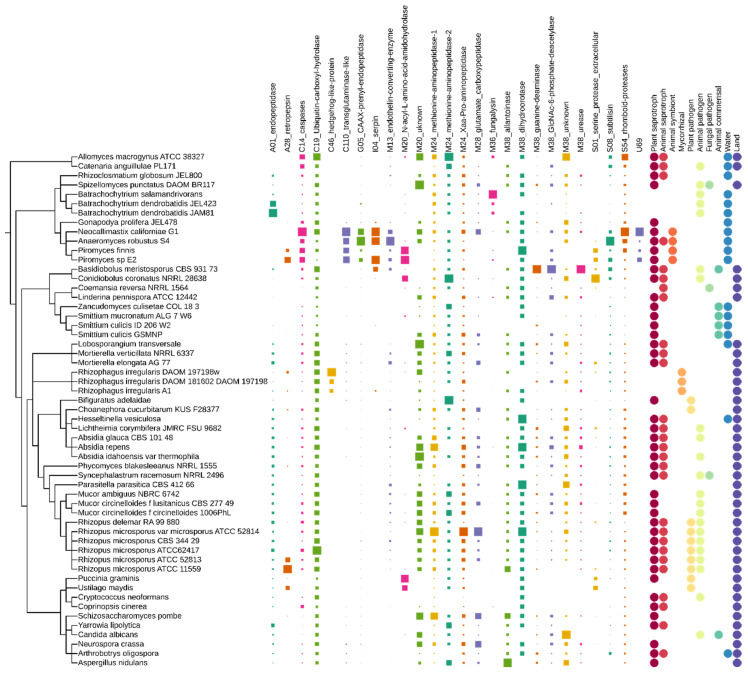
Distribution of adaptasome peptidases within early-diverging fungi. The presence of proteins belonging to a particular family is marked with squares of size corresponding to their copy number. Fungal ecology features are shown as circles.

The adaptasome encompasses nineteen MEROPS families of peptidases with twenty-six different associated biological activities (Table 1, Figure 2). The contribution of identified peptidases to fungal ecology has been described only for seven of the nineteen selected protein families. Aspartyl proteases (including A01 family pepsins) and metalloproteinases M36 are well known from studies on *Batrachochytrium* sp. pathogenicity [72]. Both *B. dendrobatidis* and *B. salamandrivoras* have a reported great expansion of M36 metalloproteases compared with a free-living chytrid *Spizellomyces punctatus* [73]. Our results confirm this trend. Pepsin A01 and subtilisin S08A proteases were described as ecologically relevant for ectomycorrhizal fungi [74]. A01 proteases also show expansion in other taxa associated with plants and saprotrophic species. The lowest copy number of A01 and S08A is observed in animal-associated *Zoopagomycota* and rumen symbiotic chytrid *Neocallimastigomycota*.

Families uncharacterized in fungi were annotated functionally based on information obtained from either animal or bacterial models. The function of two families, M20 and M38, remains fully unknown—searches against protein annotation databases (pfam, orthodb, uniprot, and blast) provide information solely on the assignment to a given MEROPS family.

#### 3.2.1. Aspartic Peptidases

We identified two families of aspartic peptidases in the adaptasome: A01 and A28. Protein domain analysis showed that aspartyl peptidases from the A28 family are unannotated retro peptidases responsible for the cleavage of the LTR retrotransposon *pol* polyprotein. Retroviral aspartic peptidases have been previously reported as problematic in sequence-based classification [75], many of them belonging to A2 and A11 families. The A28 family has not been annotated in regard to retropepins so far. These proteins are present in great copy numbers in diverse taxonomic groups (*Neocallimastigomycota*, *Glomeromycotina*, *Mucoromycotina*, *Dikarya*) (Figure 2). They show the greatest expansion in fungi associated with plants, consistent with previous reports on LTR retrotransposon abundance [76]. The presence of transposable elements in our result may be another indication that TE play a role in the adaptation of fungi to the environment [77]. It is also plausible that retropepins were domesticated and gained novel functions. On the other hand, the presence of retropepsins in the proteome can be a consequence of inefficient transposon masking prior to gene calling. We found that the number of retropepsins in the proteome does not correlate with the number of identified LTR retrotransposons in the genome.

#### 3.2.2. Cysteine Peptidases

Three families of cysteine peptidases belong to the adaptasome. Family C14 groups various caspases. Caspases are involved in programmed cell death (PCD). In fungi, PCD has multiple manifestations. This process takes place during reproduction and aging, but it is also a response to contact with some environmental conditions, such as non-self-recognition or stress [78]. The ancient fungal lineages Neocallimastocomycotina and Blastocladiomycota show the highest copy numbers of C14 caspases in our dataset.

Ubiquitin carboxyl hydrolases C19 are present in all studied fungal proteomes, including the product of *creB* gene in *Aspergillus nidulans*. This peptidase is a part of the regulatory network controlling carbon source utilization, and also plays a role in the response to carbon starvation [79,80]. In *Trichoderma reesei, creB* was proven to be associated with cellulase expression [81]. Ubiquitin carboxyl hydrolases C19 were particularly abundant in aquatic EDF and *Mucromycota*.

Homologs of family C46 show huge expansion in *Glomeromycotina* and do not occur in any other of the analysed fungi. These proteins share a common architecture of two domains: AIG1 (PF04548) and Hint (PF01079), as described previously [82]. *R. irregularis* C46 peptidases are believed to be involved in the putative mating response, which was studied in planta [82]. The Hint domain is also an element of inteins—mobile, self-splicing elements that interrupt proteins. Hint performs autoproteolytic reactions, having activity at the N- or C-terminus of an intein. Hint inteins were found both in metazoans and *Amorphea*, *Archeaplastida*, *Cryptista*, and SAR. [83].

The C110 family contains homologs of proteins coded by *Schizosaccharomyces pombe* gene *png1*. These enzymes belong to the peptide:N-glycanases responsible for the deglycosylation of misfolded glycoproteins in a system called endoplasmic-reticulum-associated degradation (ERAD) [84]. C110 proteins are especially numerous in *Neocalimastigomycota*, which are known to have heavily glycosylated CAZymes [85].

#### 3.2.3. Glutamic Peptidases

Proteins from family G05 (previously M79) show similarity to type II CAAX prenyl endopeptidase. Prenylation is the process of the addition of hydrophobic molecules to a protein. The prenyl group is attached to the CAAX motif in protein, which is crucial for the function and membrane targeting of many eukaryotic proteins [86]. These peptidases are present in all studied fungi, but are more copious in anaerobic chytrids.

#### 3.2.4. Inhibitors

Serpins (I04) constitute the single family of peptidase inhibitors in the adaptasome. Serpins are present mostly in older taxa, such as *Neocalimastigomycota* and *Entomophthtoromycotina*. These proteins have two protein domains, CBM_10 and Serpin. Inhibitors with such domain architecture were described in anaerobic fungi *Piromyces* and called celpins (from “cellulosomal seprin”). Celpins probably protect the cellulosome against proteolytic degradation by proteinases from the surrounding environment (herbivore digestive track and coexistent bacteria). Celpins were observed in the cellulosome-producing thermophilic bacterium *Clostridium thermocellum* [87]. This observation points at a likely history of the horizontal acquisition of these genes by anaerobic fungi ancestors.

#### 3.2.5. Metallo Peptidases

Peptidases from family M13 occur in all fungal taxa and are predicted to be secreted. They have the biggest expansion in anaerobic chytrids and fewer copies in *Zoopagomycota* and *Dikarya*. Bacterial M13 metallopeptidases are endopeptidases with broad substrate specificity. Taken together, these proteins probably play a nutritional role [88].

Two groups of homologs in family M20 were recognized, forming two separate clades on the phylogenetic tree. The first clade contains homologs of Aminoacylase-1, coded in humans by the *acy1* gene. Acy1 plays a role in the breakdown of acylated amino acids generated during protein degradation. In bacteria, homologs of Acy1 are implicated in the biosynthesis of arginine, lysine, and components of the cell wall [89]. Homologs from the second clade do not show a similarity to characterized proteins.

Family M24 groups peptidases with different molecular targets, which function as: methionine aminopeptidases (MAP1/MAP2 in *Saccharomyces cerevisiae*) and Xaa-Pro aminopeptidase. MAP1 and MAP2 are known to play an essential role in the maturation of a nascent polypeptide during translation. The deletion of *map1* and *map2* genes in yeast is lethal [90]. Other homologs from this family show similarity to Xaa-Pro aminopeptidase pepP from *Aspergillus nidulans*. In animals, this enzyme is involved in the utilization of bradykinin [91]. In fungi, these proteins probably play a role in nutrition. In our study, MAP1 was especially abundant in *Absidia* spp. and *Schizosaccharomyces pombe*. MAP2 occurs in the greatest copy number in *Blastocladiomycota*, *Conidiobolus coronatus*, and *Endogonales*. Proteins with Xaa-Pro-aminopeptidase activity seem to be more common in *Mucorales* than in other taxa.

We identified M28 peptidases in almost all taxa with the exclusion of *Glomeromycotina* and *Basidiomycota*. However, the function of these proteins remains unknown in fungi. The most similar proteins to our sequences are glutamate carboxypeptidase II (GCPII) and transferrin receptor protein 1. GCPII has been well studied for human and mouse homologs. In humans, this enzyme is expressed predominantly in the central nervous system—where it hydrolyzes one of the neurotransmitters—in the prostate, small intestine, and kidney [92]. Transferrin is one of the key proteins to regulate iron homeostasis in vertebrate cells [93].

Metalloproteinases M38 are particularly abundant in *Blastocladiomycota*, chytrids, *Zoopagomycota*, and *Mucoromycota*. This family has been divided into subfamilies and our results contain six subfamilies. Four of them are related to the uric acid degradation pathway. Urease has conserved roles in promoting fungal infections. It was shown that urease is required for the full virulence of *Cryptococcus neoformans* and *Coccidioides posadasii* in animal models. The activity of urease during fungal infection is important in multiple ways: it is linked with providing nitrogen for the pathogen, and urea is metabolized to ammonia which takes part in damaging host tissues and interfering in the functions of phagocytes [94]. The activity of urease in fungal infections led to the development of urease tests for diagnostic purposes. We observed the greatest abundance of urease in *Basidiobolales*. The remaining two subfamilies are amidohydrolases with unknown functions and proteins similar to *N*-acetylglucosamine 6-phosphate deacetylase (nagA). This enzyme takes part in utilizing a carbon source, *N*-acetylglucosamine (GlcNAc), which is a major component of structural polymers in bacteria, plants, and animals. GlcNAc is also known to have a stimulatory effect on filamentation in Ascomycota [95]. In our study, they were mostly abundant in *Blastocladiomycota*, *Basidiobolales*, and *Mucoromycota*.

#### 3.2.6. Serine Peptidases

The adaptasome includes tree serine proteases families: S01 (chymotrypsin-like), S08 (subtilisin), and S54 (rhomboid). Relationships between these proteins and fungal ecology were previously described for pathogenic [96] and saprotrophic lifestyles [97]. Next, researchers confirmed the high abundance of S01 proteins in the proteomes of pathogenic fungi and S08 in proteomes of soil/dung fungi [98]. Our result also indicates a high occurrence of S08 homologs in anaerobic fungi—it may be due to environmental requirements in the herbivore digestive tract. The rhomboid serine protease (S54) family includes yeast proteins Pcp1/Rbd1 and Rbd2. Members of the rhomboid family were divided into five subfamilies of which RhoA, RhoD and PARL are present in fungi. Their number was shown to correlate positively with a pathogenic lifestyle in fungi [98], and consistent with the prediction, *A. fumigatus* RbdB was critical for virulence [99].

#### 3.2.7. Unknown Peptidases

Unknown family U69 groups CAZymes rather than peptidases. A representative of this family, Cthe_2159 from *Clostridium thermocellum*, has been characterized structurally, showing similarities with polysaccharide lyases. It is also the first representative of a novel family of cellulose and/or acid-sugar binding beta-helix [30]. This observation shows that protein family classification is an ongoing process, with reclassifications happening constantly.

### 3.3. Transporters

**Figure 3:**
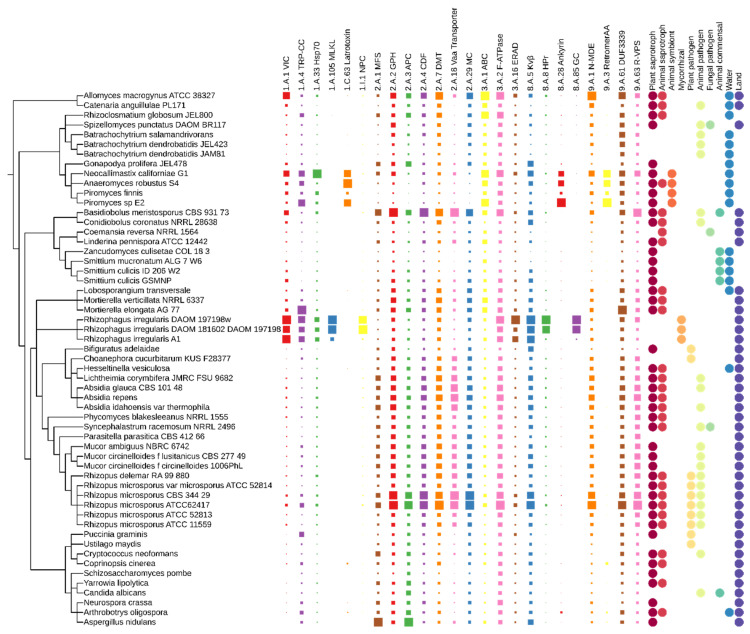
Distribution of adaptasome transporters within early-diverging fungi. The presence of proteins belonging to a particular family is marked with squares of size corresponding to their copy number. Fungal ecology features are shown as circles.

Twenty-four classes of transporters belong to the adaptasome. TCDB proteins show four characteristic distribution patterns. The first one includes expansions of families involving essential transporters such as MSF (major facilitator superfamily), DMT (drug/metabolite transporters), transporters associated with the uptake of nutrients, ion channels and others, covering most of the analysed fungal lineages (Figure 3). These transporter classes are particularly abundant in *Mucoromycota* and *Basidiobolales*. The second pattern can be defined as *Glomeromycotina*-specific expansions of classes: mixed lineage kinase domain-like, nuclear pore complex, phosphotransferase system HPr, and guanylate cyclases. The third pattern is limited to anaerobic chytrids—these are families with an ankyrin domain. The last group of transporter expansions happened in the mutualists *Neocallimatigomycotina* and *Glomeromycotina*—VIC (voltage-gated ion channels), TRP-CC (the transient receptor potential Ca^2+^ channels), and Hsp70. The interpretation of the transporter constituents of the adaptasome is highly non-trivial due to the limited knowledge of transporters overall. TCDB often describes a particular copy of a given transporter in a very detailed cell/tissue/structure which is not generalizable or even transferable from human to fungi. It is already known that transporters and receptors can be recruited to highly diverged and sophisticated roles. In this section, we will summarize what is known on the transporter families, starting from the best studied and characterized in fungi to the least recognized in the literature.

#### 3.3.1. Essential Transporters

Not surprisingly, the adaptasome encompasses the most common transporters, crucial for the functioning of organisms, such as MFS, ABC, DMT, VPS and other vacuolar transporters. Fungal homologs of these transporters have documented roles in drug resistance and nutrition. They were particularly abundant in *Blastocladiomycota*, chytrids, *Basidobolales*, and *Mucoromycota* and had a limited number of homologs in *Dikarya* and *Kickxellomycotina*.

The major facilitator superfamily (MFS) (2.A.1) are ubiquitous membrane proteins with millions of sequenced members. The MFS family was originally believed to function primarily in the uptake of sugars, but subsequent studies revealed that drugs, metabolites, oligosaccharides, amino acids and oxyanions were all transported by MFS family members [100]. In fungi there are known MFS transporters responsible for the transport of glucose [101], fructose [102], galactose and lactose [103], maltose [104], cellobiose [105] and more. Taken together, MFS transporters are essential for fungal nutrition. EDF show expansions of this kind of transporter, especially in rapidly growing sugar fungi *Basidiobolales* and *Mucoromycota*.

The ATP-binding cassette (ABC) superfamily (3.A.1) is one of the largest and possibly one of the oldest gene families. These ubiquitous transporters are more commonly closer to the root of FToL—in *Blastocladiomycota* and chytrids, but also in *Mortierella* spp. ABC transporters have been characterized from both prokaryotes and eukaryotes; they are essential for many processes in the cell [106]. There are many examples of characterized fungal ABC transporters: Mdr1 protein, responsible for *Aspergillus fumigatus* drug resistance [107]; *S. cerevisiae* Pxa1, involved in the import of long-chain fatty acids from cytosol to peroxisomes; Pdr18 that inserts ergosterol into the membrane; vacuolar metal resistance and drug resistance protein Ycf1 [108]; and more.

The drug/metabolite transporter (DMT) superfamily (2.A.7) is a complex ensemble of 31 families, and fungal homologs are present in 11 of them (TCDB website info). DMT are abundant in *Blastocladiomycota*, *Chytridiomycota*, and *Mucoromycota*. The triose-phosphate transporter (TPT) family (2.A.7.9) members are derived from the inner envelope membranes of chloroplasts and non-green plastids of plants. However, homologs are also present in yeast, but their function is unknown [109]. The UDP-*N*-acetylglucosamine: UMP antiporter (UAA) family (2.A.7.10) proteins are found in the Golgi apparatus and the endoplasmic reticulum of eukaryotic cells. Yea4 protein from *S. cerevisiae* is a sugar transporter required for cell wall chitin synthesis [110]. The same in-cell localization is characteristic for the UDP-galactose: UMP antiporter (UGA) family (2.A.7.11). Hut1 *S. cerevisiae* protein from this family is involved in the maintenance of an optimal environment for the folding of secretory pathway proteins in the endoplasmic reticulum [111]. The GDP-mannose:GMP antiporter (GMA) family (2.A.7.13) includes fungal transporters involved in hyphal formation, e.g., Vrg4 from *C. albicans* [112]. Fungal proteins were also observed in family 2.A.7.16 (the GDP-fucose transporter (GFT) family) [113]. The thiamine pyrophosphate transporter (TPPT) family (2.A.7.24) includes yeast protein Thi74, which may be involved in thiaminediphosphate transport across the mitochondrial membrane [114]. The NIPA Mg^2+^ uptake permease (NIPA) family (2.A.7.25) groups proteins acting as Mg^2+^ transporters, which can also transport other divalent cations such as Fe^2+^, Sr^2+^, Ba^2+^, Mn^2+^ and Co^2+^ but to a much lesser extent than Mg^2+^. This family includes two uncharacterized fungal homologs [115]. The Ca^2+^ homeostasis protein (Csg2) family (2.A.7.27) has only one described protein, Csg2 from *S. cerevisiae*. Csg2 is required for calcium regulation—it may regulate the calcium level by a non-vacuole organelle. It is worth noticing that this protein also plays a role in ceramide synthesis [116]. The solute carrier 35G (SLC35G) family (2.A.7.28) includes one fungal homolog which contains the HeLo (PF14479) domain. HeLo is known for a prion-inhibitory effect and is found exclusively in the kingdom *Fungi* [117]. Bacterial/archaeal transporter (BAT) family (2.A.7.2) includes two uncharacterized fungal sequences. The last family containing fungal homologs is the uncharacterized DMT-4 (U-DMT4) family (2.A.7.32)—the function of the included proteins remains unknown.

The retromer-dependent vacuolar protein-sorting (R-VPS) family (9.A.63) has several expansions in *Basidiobolales* and *Mucoromycota*. The retromer complex is a trafficking assembly composed of at least three proteins—Vps26, Vps29 and Vps35—in *Saccharomyces cerevisiae*. Proteins from this family play a role in the intracellular sorting and delivery of soluble vacuolar proteins [118]. The VPS system in fungi is one of the main routes of vesicle formation, essential for autophagy and pathogenesis [119,120].

Family 2.A.18 (vacuolar amino acid transporter) include fungal amino acid permeases, e.g., PcMtr protein from *Penicillium chrysogenum*. These proteins are not limited to Dikarya and are found abundantly in *Basidiobolales* and *Mucoromycota*. Fungal permeases are responsible for the uptake of amino acids, which can serve as a nitrogen and carbon source. They may also be involved—such as Mtr-type transporters—in the uptake of signalling molecules [121].

#### 3.3.2. Less Frequent Fungal Transporters

Transporters with some characterized fungal homologs. These families are essential transporters such as VIC, ion channels, nuclear pore complexes, transporters involved in the uptake of nutrients, mitochondrial carriers, drug transporters, and others.

The voltage-gated ion channel (VIC) superfamily (1.A.1) occurs abundantly in *Neocallimastigomycota* and *Glomeromycotina*. There are two described VIC proteins in fungi, both from *Saccharomyces cerevisiae*: TOK1, which is an outwardly rectifying K+ channel with a unique structure and function [122], and calcium channel protein Cch1, which is essential for a full response to ion stress [123].

The transient receptor potential Ca^2+^ channel (TRP-CC) family (1.A.4) proteins are involved in the response to changes in the environment and seem to be duplicated in the symbiotic fungi *Neocallimastigomycota* and *Glomeromycotina*. Calcium ions, present inside all eukaryotic cells, are important second messengers in the transduction of biological signals. Four proteins from this family were described in yeast. YVC1 protein is required for the release of calcium ions from the vacuole during hyperosmotic shock [124]. FLC1 and FLC2 (Flavin carrier protein 1/2) probably play a role in oxidative protein folding [125]. PKD2 protein is a key signalling component in the cell wall synthesis [126]. Therefore, their expansion in organisms closely interacting with plants (Glomeromycota) or animals (Neocallimastigomycota) is not surprising.

Family 1.I.1—the nuclear pore complex (NPC) family (formerly 1.A.75)—contains proteins with a variety of domain architectures, including proteins with domains from PKinase (CL0016) clan. As homologs of this family are present in outstanding numbers in *Glomeromycotina*, it is worth noting that these fungi show unusual cell organisation of their cells with multiple coexisting nuclei labelled as exhibiting polykaryosis [127].

The glycoside–pentoside–hexuronide (GPH):cation symporter family (2.A.2) catalyse the uptake of sugars and are present in all analysed taxa, with the greatest expansion in *Basidiobolales* and *Mucoromycota*. General alpha-glucoside permease sut1 from *S. cerevisiae* is responsible for the transport of maltose and sucrose into the cell [128]. This finding may be considered as a potential mechanism underlying the effective growth of Mucoromycota representatives on maltose and sucrose [50].

The mitochondrial carrier (MC) family (2.A.29) proteins transfer molecules across the membranes of the mitochondria. This family has a number of known fungal homologs and in our study is particularly abundant in *Blastocladiomycota*, *Basidiobolales*, and *Mucoromycota*. Among them are proteins that mediate the import of ADP into the mitochondrial matrix for ATP synthesis [129], dicarboxylic transporters catalysing the exchange of dicarboxylic acids such as malate and succinate for inorganic phosphate [130], proteins that transport FAD from the cytosol to the mitochondrial matrix [131].

The amino acid–polyamine–organocation (APC) superfamily (2.A.3) in our study is present in all studied fungi and has the greatest expansions in *Basidiomycota* and *Mucoromycota*. At least three APC subfamilies were experimentally characterized in fungi. The amino acid/choline transporter subfamily (2.A.3.4) includes transporters from fungi and *Actinobacteria*. Among them are γ-aminobutyric acid (GABA) permease and 7-keto-8-aminopelargonic acid (KAPA) transporter, and vitamin B1 transporter. The L-type amino acid transporter (LAT) subfamily (2.A.3.8) includes fungal L-methionine transporter. Histidin, proline, arginine, glutamine, tryptophan, lysin, and leucine permeases are examples of proteins from subfamily 2.A.3.10 (the yeast amino acid transporter (YAT)) [113,132].

The cation diffusion facilitator (CDF) family (2.A.4) proteins transport heavy metals including cobalt, cadmium, iron, zinc and possibly nickel, copper and mercuric ions. There are known fungal mitochondrial, vacuolar and nuclear permeases [133]. Fungi are famous for their resistance to metal ions. In our study, CDF transporters are commonly present in *Basidiobolales* and *Mucoromycota*.

The endoplasmic reticular retrotranslocon (ER-RT or ERAD) family (3.A.16) are responsible for the translocation of misfolded protein from the lumen of the endoplasmic reticulum to cytoplasm, where they are degraded (e.g., Ssm4 from *S. cerevisiae* [134]). We noticed a huge expansion of ERAD translocons in *Glomeromycotina*.

The H^+^- or Na^+^-translocating F-type, V-type and A-type ATPase (F-ATPase) superfamily (3.A.2) was found in all studied taxa, but it is most abundant in plant-associated EDF. These enzymes catalyse the decomposition of ATP to ADP. They are necessary for cell metabolism—the export of toxins, wastes and solutes that may hinder cellular process. F-type ATPases are found in eukaryotic mitochondria and chloroplasts, as well as in bacteria. V-type ATPases are found in the vacuoles of eukaryotes and in bacteria. A-type ATPases are found in archaea. [135].

The non-ABC multidrug exporter (N-MDE) family (9.A.1) was observed most commonly in *Blastocladiomycota* and *Mucoromycota*. The N-MDE family includes two fungal proteins with uncharacterized function: beta-chimaerin from *Microsporum canis* (C5FZG4) and Ncu02524 from *Neurospora crassa* (Q7SHT9).

#### 3.3.3. Uncharacterized Fungal Transporters

The last group of transporters are proteins without characterized fungal homologs. For some of them, one can infer function on the basis of characterized proteins, e.g., from *Metazoa* or *Bacteria*. There are also proteins with neither known function in any branch of the tree of life nor with functions which have translation to fungi.

The mixed lineage kinase domain-like (MLKL) family (1.A.105) is characterized in humans. MLKL protein is a pseudokinase that plays a role in programmed cell death [136]. We found homologs of this family exclusively in *Glomeromycotina*. They possess a repetitive Sel1 domain (PF08238) with no clear function in fungi.

Family 1.A.33 (the cation channel-forming heat shock protein-70 (Hsp70) family) was noticed mostly in anaerobic chytrids and *Glomeromycotina*. Hsp70 representatives are molecular chaperones that are often associated with membranes. Hsp70 proteins are well known in humans. These proteins are implicated in a variety of cellular processes, including the protection of the proteome during stress, the folding and transport of newly synthesized proteins, and the activation of misfolded protein lysis [137].

Repeat-bearing protein families with ankyrin domains are expanded in *Neocallimastigomycota*. Such repeats were documented in diverse fungal proteins, including Yap-related transcription factors [138] and NUC-2 protein [139]; however, studies on the role of these repeats in fungi are missing. Family 1.C.63 is known as the α-latrotoxin (latrotoxin) family. The α-latrotoxin family in TCDB includes solely Mediterranean black widow spider proteins [140] with Ank_2 (PF12796). The latter Ank_2 domain may play a role in protein folding [141]. The role of 1.C.63 in fungi is elusive. Additionally, the main ankyrin (ankyrin) family (8.A.28) does not include characterized fungal proteins. It is known that ankyrin repeats are involved in mediating protein–protein interactions [141].

The voltage-gated K+ channel β-subunit (Kvβ) family (8.A.5) has no characterized fungal homologs, but we found it in all studied taxa. It has the greatest expansion in *Glomeromycotina*. Rat protein Kcnab1 is a cytoplasmic subunit that promotes pore-forming by the channel-forming alpha-subunits [142].

The phosphotransferase system HPr (HPr) family (8.A.8) consists of bacterial proteins, all with the function of phosphoryl transfer protein. However, we found HPr homologs in several fungi, with a great number of copies in *Glomeromycotina*. ptsH protein from *Escherichia coli* is a major component of phosphoenolpyruvate-dependent sugar phosphotransferase system (sugar PTS). This carbohydrate active-transport system catalyses the phosphorylation of incoming sugar substrates concomitantly with their translocation across the cell membrane [143].

The guanylate cyclase (GC) family (8.A.85) controls many cellular processes from growth viability, and differentiation to contractility, secretion, and ion transport [144]. There are no described fungal guanylate cyclases, although in our analysis it turned out to be highly abundant in *Glomeromycotina*. Human and *Bacteria* characterized guanylate cyclases do not provide the grounds to infer about the function of this family in fungi.

The retromer assembly apparatus (RetromerAA) family (9.A.3) were detected in *Neocallimastigomycota*. The closest characterized RetromerAA homolog is the Snx27 human protein. It is involved in the trafficking pathway that promotes the recycling of internalized transmembrane proteins—proteins are delivered to the early endosome and recycled to the plasma membrane instead of being degraded in lysosomes [145]. It is an open question as to whether the molecular function was kept by all *Opisthokonta* descendants, including fungal homologs.

### 3.4. Protein Expansions and Ecology

Expansions and losses observed in the sets of CaZymes, peptidases and transporters altogether reveal a landscape of adaptive combinations. One of the most illustrative examples comes from *Neocallimastigomycota*, anaerobic fungi which show a great abundance of CAZymes responsible for the degradation of organic plant matter such as xylan/pectin esterases, cellulases, and glucanases in numbers far greater compared with other plant-associated fungi. *Neocallimastigomycota* also show unique expansions of many peptidases: C110, G05, I04, M13, M20, S54, S08, C14, U69. The biological function of most of these peptidases and inhibitors still remains to be described, which prevents discussing their relevance to fungal ecology in more detail. In the case of transporters, anaerobic fungi show expansions of the ankyrin family, and the latrotoxin family (of yet unknown function in fungi). They share some expansion patterns with other symbionts such as arbuscular mycorrhizal fungi—both fungal groups have numerous channels involved in the stress response, such as Hsp70, VIC and TRP-CC (1.A.4). Glomeromycota additionally have more essential MLKL transporters and an ERAD transport system (associated with the degradation of misfolded protein) than other fungi. It has been shown before that mycorrhizal fungi possess several characteristic protein family contractions and losses involving plant-biomass-degrading enzymes [146]. However, in mycorrhizal systems the transporters have been described more thoroughly on the plant’s side, leaving fungal counterparts uninvestigated [147].

Plant-associated *Mucoromycota* display a recursive expansion of the protein families involved in nutrition, drug and heavy metal resistance. However, not only nutrition-related CaZymes tend to be duplicated. Adaptasome CaZyme families are more often related to cell wall remodelling than to plant biomass degradation. This observation is in line with the role of the fungal cell wall as a key compartment crucial for the adaptation to a particular niche and stress response element [55,148]. Animal-associated fungi have a range of expansions related to pathogenicity and nutrition, providing a clear link between the host type and enzymes involved; peptidases for vertebrate parasites and chitinases for fungi living with invertebrates. Amphibian pathogens *Batrachochytrium* spp. have a much higher abundance of endopeptidase A01 and fungalysin than other fungi. Fungi which live with arthropods (as pathogen or commensal) have characteristic patterns of CAZymes—they have enzymes such as chitinases, specific kinds of glucanases, glucosidases and benzoquinone reductases. This is consistent with previous reports on protein family evolution in amphibian pathogens [149], dermatophytes [150] and entomopathogenic fungi [151].

## 4. Conclusions

The main goal of this study is to provide a rational explanation of how the presence of particular proteins shapes the adaptability of early-diverging fungi. Almost half of the protein families identified in our study as highly variable among EDF lineages have never been characterized in fungi and have no clear assignment of function. The observed distribution of several protein families with an unknown role in fungi underscores the ancestral inheritance shared with the remaining *Opisthokonta* lost in Dikarya. These protein families are not only present but also numerous and expressed in diverse isolates. These results point at an urgent need for experimental studies aimed at the functional characterization of adaptasome proteins. Particular homologs may display different regulatory mechanisms, substrate specificity and interacting partners in the biological context of individual strains or lineages.

We found that protein families, which have previously attracted attention, have dynamic evolutionary histories reflecting their adaptive potential towards new ecological challenges. Among others, ABC and MFS transporters, which enable resistance against multiple environmental threats [152,153], are proteins involved in fungal cell wall biosynthesis such as chitin synthases (GT2) essential for interactions with other organisms and fungal defence [47]. Nutritional mode has a profound impact on the evolution of fungal genomes and the majority of differentially represented protein families in fungal proteomes are indeed associated with the digestion of nutrients (e.g., chitinases GH19 in fungi living with insects and loss of acetyl-xylan esterases CE2 and CE6 in endomycorrhizal Glomeromycota). The adaptasome encompasses a number of proteins involved in the modification of proteins which likely contribute to orchestrated protein regulation.

We also demonstrate a correlation between the adaptasome and ecological niches. This is particularly pronounced for anaerobic fungi and their outstanding repertoire of enzymes, enabling them to survive in atypical conditions. *Neocallimastigomycota* fungi may be classified as plant associated, and also as animal associated, but their family distribution patterns differs from patterns characteristic to both categories. Anaerobic fungi show an abundance of plant-degrading enzymes [154] and simultaneously unique expansions of peptidases and transporters. However, it is hard to determine which protein families are most relevant to the peculiar niche occupied by the anaerobic fungi since the precise biological function of many of these expanded protein families remains to be described. Neocallimastigomycota and other symbionts, such as arbuscular mycorrhizal fungi, are characterized by numerous channels involved in the stress response. One might speculate whether these channels have been co-opted to the symbiotic lifestyle and have a role in maintaining the relationship with the host.

Early-diverging fungi adopt a remarkable diversity of lifestyles and simultaneously are broadly sequenced, which makes them a good system to study genome–adaptability relationships. They share multiple common proteins with non-Dikarya opisthokonta, which raises many questions about their metabolism and ecology. The identified list of protein families provides a likely roadmap for the experimental phenotype screening of EDF and perhaps other fungal lineages. Some protein classes identified in this study were characterized exclusively in higher eukaryotes in specialized tissues, obviously not present in fungi. A more comprehensive understanding of the biology of EDF might provide invaluable insight into the key events in the evolution of life.

## Figures and Tables

**Table 1:** Summary of protein families with statistically significant changes in copy number distribution among early-diverging fungi (EDF). Functional annotations were based on UniProt, protein domain role and literature searches.

MEROPS		
MEROPS ID	Predicted Name	Putative Role in Fungi
A01	endopeptidase	Nutrition, pathogenicity
A28	retropepsin	Unknown
C110	peptide-N(4)-(*N*-acetyl-β-glucosaminyl) asparagine amidase	Protein degradation
C14	caspases	Controlled cell death
C19	ubiquitin-carboxyl-hydrolase	Nutrition
C46	hint domain containing	Putative mating response
G05	type II CAAX-prenyl-endopeptidase	Protein modification
I04	serpin	Peptidase inhibitor
M13	unknown peptidase	Nutrition
M20	*N*-acyl-l-amino-acid-amidohydrolase	Nutrition
	unknown peptidase	Unknown
M24	MAP1	Maturation of the nascent polypeptide during translation
	MAP2	
	Xaa-Pro aminopeptidase	Nutrition
M28	glutamate carboxypeptidase	Unknown
M36	fungalysin	Nutrition, pathogenicity
M38	allantoinase	Uric acid degradation pathway, pathogenicity
	unknown peptidase	
	dihydroorotase	
	guanine deaminase	
	urease	
	*N*-acetylglucosamine-6-phosphate-deacetylase	Chitin degradation
S01	serine protease (extracellular)	Nutrition
S08	subtilisin	Nutrition
S54	rhomboid proteases	Mitochondrial endopeptidase
U69	polysaccharide lyases	Nutrition
**CAZY**		
**CAZY ID**	**Predicted Name**	**Putative Role in Fungi**
AA3	glucose-methanol-choline (GMC) oxidoreductases	Nutrition
AA5	galactose oxidase	Nutrition
AA6	1,4-benzoquinone reductase	Nutrition, interaction with insects
AA7	glucooligosaccharide-oxidase/chitooligosaccharide-oxidase/cellooligosaccharide-dehydrogenase	Cell wall remodelling
CE1	esterase domain containing	Nutrition
	feruloyl/acetylxylan esterase	Nutrition
	rhamnogalacturonan-acetylesterase	Nutrition
CE12	rhamnogalacturonan-acetylesterase	Nutrition
CE16	acetylesterase	Nutrition
CE2	acetyl-xylan esterase	Nutrition
CE4	chitin deacetylase	Cell wall remodelling
CE6	acetyl-xylan esterase	Nutrition
GH10	xylanase	Nutrition
GH109	α-*N*-acetylgalactosaminidase	Glycolipids modification
GH11	endo-1-4-β-xylanase	Nutrition
GH114	α-galactosidase	Nutrition
GH132	β-glucosidase	Pathogenicity
GH15	glucoamylase	Nutrition
GH16	endo-β-glucanase	Nutrition
GH17	glucan-1,3-β-glucosidase	Cell wall remodelling
GH18	chitinase	Cell wall remodelling
GH19	chitinase	Interaction with insects
GH24	lysozyme	Cell wall remodelling
GH26	endo-1,4-β-mannosidase	Cell wall remodelling, nutrition
GH3	β-glucosidase	Nutrition
GH43	arabinase/levansucrase/invertase	Nutrition
GH45	endoglucanase	Nutrition
GH48	cellulase	Nutrition
GH5	cellulase	Nutrition
	exo-β-1,3-glucanase	Cell wall remodelling
GH6	cellobiohydrolase	Nutrition
	endoglucanase	Nutrition
GH72	1,3-β-glucanosyltransferase	Cell wall remodelling
GH9	cellulase	Nutrition
GT1	UDP-glucuronosyltransferase	Cell wall remodelling
GT15	α-1,2-mannosyltransferase	Cell wall remodelling, pathogenicity
GT17	β-1,4-mannosyl-glycoprotein-β-1,4-*N*-acetylglucosaminyltransferase	Cell wall remodelling
GT2	chitin synthase 2	Cell wall remodelling
	chitin synthase 6	Cell wall remodelling
GT34	galactomannan-α-1,6-galactosyltransferase/xyloglucan-α-1,6-xylosyltransferase/α-1,2-galactosyltransferase	Cell wall remodelling
GT49	β-1,3-*N*-acetylglucosaminyltransferase	Glycosylation
GT68	GT68_protein-*O*-α-fucosyltransferase	Protein modification
GT69	α-1,3-mannosyltransferase	Cell wall remodelling, pathogenicity
GT71	α-mannosyltransferase	Cell wall remodelling, protein modification
GT77	α-xylosyltransferase/α-1,3-galactosyltransferase/arabinosyltransferase	Protein modification
PL1	pectin lyase	Nutrition
PL3	pectate lyase	Nutrition
PL4	rhamnogalacturonan endolyase	Nutrition
**TCDB**		
**TCDB ID**	**Family Name**	**Putative Role in Fungi**
1.A.1	The Voltage-gated Ion Channel (VIC) Superfamily	Response to stress
1.A.105	The Mixed Lineage Kinase Domain-like (MLKL) Family	Programmed cell death
1.A.33	The Cation Channel-forming Heat Shock Protein-70 (Hsp70) Family	Response to stress
1.A.4	The Transient Receptor Potential Ca^2+^ Channel (TRP-CC) Family	Response to stress
1.C.63	The α-Latrotoxin (Latrotoxin) Family	Unknown
1.I.1	The Nuclear Pore Complex (NPC) Family [formerly 1.A.75]	Unknown
2.A.1	The Major Facilitator Superfamily (MFS)	Nutrition, drug and metabolites transport
2.A.18	Vacuolar amino acid transporter	Nutrition
2.A.2	The Glycoside-Pentoside-Hexuronide (GPH): Cation Symporter Family	Nutrition
2.A.29	The Mitochondrial Carrier (MC) Family	Molecules transfer to mitochondria
2.A.3	The Amino Acid-Polyamine-Organocation (APC) Superfamily	Nutrition
2.A.4	The Cation Diffusion Facilitator (CDF) Family	Heavy metal transport
2.A.7	The Drug/Metabolite Transporter (DMT) Superfamily	Drug/ion resistance, cell wall remodelling
3.A.1	The ATP-binding Cassette (ABC) Superfamily	Essential for many processes in the cell
3.A.16	The Endoplasmic Reticular Retrotranslocon (ER-RT or ERAD) Family	Protein degradation
3.A.2	The H+- or Na+-translocating F-type, V-type and A-type ATPase (F-ATPase) Superfamily	Decomposition of ATP to ADP
8.A.28	The Ankyrin (Ankyrin) Family	Unknown
8.A.5	The Voltage-gated K+ Channel β-subunit (Kvβ) Family	Unknown
8.A.8	The Phosphotransferase System HPr (HPr) Family	Unknown
8.A.85	The Guanylate Cyclase (GC) Family	Unknown
9.A.1	The Non ABC Multidrug Exporter (N-MDE) Family	Drug resistance
9.A.3	The Retromer Assembly Apparatus (RetromerAA) Family	Protein recycling
9.A.63	The Retromer-dependent Vacuolar Protein Sorting (R-VPS) Family	Intracellular sorting

## Data Availability

The analyses are based on publicly available sequences. All identifiers of analysed proteins are listed in the Supplementary Materials. All trees built from those sequences are also listed in the Supplementary Materials.

## Supplementary Materials

The following supporting information can be downloaded at: www.mdpi.com/article/10.3390/jof8010067/s1, Supplementary Table S1: Table with numbers of homologs per adaptasome family in all studied non-Dikarya fungi. Supplementary Table S2: Table with information about the ecology of the analysed fungi derived from the literature. Supplementary Table S3: Accessions of all identified protein homologs. Supplementary Table S4: PMIDs of sources of information about all studied genomes. Supplementary Dataset DS1: File with phylogenetic (ML) trees of all studied protein families in non-Dikarya fungi in Newick format.
